# A microRNA Cluster-Lefty Pathway is Required for Cellulose Synthesis During Ascidian Larval Metamorphosis

**DOI:** 10.3389/fcell.2022.835906

**Published:** 2022-03-15

**Authors:** Xueping Sun, Xiaoming Zhang, Likun Yang, Bo Dong

**Affiliations:** ^1^ Sars Fang Centre, MoE Key Laboratory of Marine Genetics and Breeding, College of Marine Life Sciences, Ocean University of China, Qingdao, China; ^2^ Laboratory for Marine Biology and Biotechnology, Qingdao National Laboratory for Marine Science and Technology, Qingdao, China; ^3^ Institute of Evolution and Marine Biodiversity, Ocean University of China, Qingdao, China

**Keywords:** microRNA cluster, mesenchymal cell, cellulose synthesis, *lefty*, *Ciona*, tunicates

## Abstract

Synthesis of cellulose and formation of tunic structure are unique traits in the tunicate animal group. However, the regulatory mechanism of tunic formation remains obscure. Here, we identified a novel microRNA cluster of three microRNAs, including *miR4018a*, *miR4000f*, and *miR4018b* in *Ciona savignyi*. *In situ* hybridization and promoter assays showed that *miR4018a/4000f/4018b* cluster was expressed in the mesenchymal cells in the larval trunk, and the expression levels were downregulated during the later tailbud stage and larval metamorphosis. Importantly, overexpression of *miR4018a/4000f/4018b* cluster in mesenchymal cells abolished the cellulose synthesis in *Ciona* larvae and caused the loss of tunic cells in metamorphic larvae, indicating the regulatory roles of *miR4018a/4000f/4018b* cluster in cellulose synthesis and mesenchymal cell differentiation into tunic cells. To elucidate the molecular mechanism, we further identified the target genes of *miR4018a/4000f/4018b* cluster using the combination approaches of TargetScan prediction and RNA-seq data. *Left–right determination factor* (*Lefty*) was confirmed as one of the target genes after narrow-down screening and an experimental luciferase assay. Furthermore, we showed that *Lefty* was expressed in the mesenchymal and tunic cells, indicating its potentially regulatory roles in mesenchymal cell differentiation and tunic formation. Notably, the defects in tunic formation and loss of tunic cells caused by overexpression of *miR4018a/4000f/4018b* cluster could be restored when *Lefty* was overexpressed in *Ciona* larvae, suggesting that *miR4018a/4000f/4018b* regulated the differentiation of mesenchymal cells into tunic cells through the *Lefty* signaling pathway during ascidian metamorphosis. Our findings, thus, reveal a novel microRNA-*Lefty* molecular pathway that regulates mesenchymal cells differentiating into tunic cells required for the tunic formation in tunicate species.

## Introduction

Development of Urochordate ascidians experiences a swimming larval-to-sessile juvenile metamorphic transition. During this process, part of the larval organs, such as the notochord and sensory organ disappeared, while novel adult organs, such as the heart and digestive apparatus are lately formed (Karaiskou et al., 2015; Sasakura and Hozumi, 2018; Hotta et al., 2020). It has been revealed that certain adult organs are derived from the larval mesenchymal cells (Hirano and Nishida, 1997; Imai et al., 2003; Tokuoka et al., 2005; Fodor et al., 2021).

Mesenchymal stem cells (MSCs) are stromal cells that can self-renew and exhibit multilineage differentiation, with the capability to become an important source of tissue and organ formation and establish the cardiovascular, hematopoietic, bone, and soft tissues during embryonic development (Ding et al., 2011). MSCs in vertebrates, such as humans and mice, are divided into three cell groups: mesenchymal stromal/stem cells, pericytes, and smooth muscle cells. They differentiate into multiple organs, thus, are used wildly in cell engineering and human clinical medicine (Ding et al., 2011; Slukvin and Kumar, 2018; Shin et al., 2020; Storer et al., 2020). In invertebrates, especially in ascidian *Ciona*, mesenchymal cells are derived from the A7.6, B8.5, and B7.7 cell lineages at the 110-cell stage (Imai et al., 2003; Nakamura et al., 2012). Ascidians *Halocynthia* sp. and *Ciona* sp. mesenchymal cells derived from A7.6 cells are named as trunk lateral cells (TLC) (Imai et al., 2003; Nakamura et al., 2012; Satoh, 2014), and they mainly contribute to the formation of adult blood cells, epithelial cells of the first/second branchial cleft, tunic cells, muscle cells of the inlet and outlet, body wall muscle cells, and part of the stomach (Jeffery et al., 2008). Mesenchymal cells derived from B7.5 cells are trunk ventral cells (TVC) (Nakamura et al., 2012). They migrate between the abdominal surface and the endoderm of the trunk, and eventually differentiate into cells in tissues and organs, such as the adult heart, pericardium, and body wall muscles (Hirano and Nishida, 2000; Tokuoka et al., 2005). Mesenchymal cells derived from B7.7 and B8.5 cells differentiate into the tunic and part of the stomach of the adult (Satoh, 2014). In ascidians, mesenchymal cells have conserved behaviors and property as those in vertebrates, such as migration and differentiation (Imai et al., 2003; Tokuoka et al., 2005). Thus, this simple animal is an ideal model at an evolutionary perspective to study the functions of mesenchymal cell and their associations with developmental signaling pathways.

Tunic, composed of cellulose, is a unique structure in a tunicate animal (Dehal et al., 2002). Tunic also has been revealed to be a complex and active tissue with mesenchymal properties (Matos et al., 2020). It had been shown that mesenchymal cells can migrate across epidermal cells into tunic (Cloney and Grimm, 1970; Di Bella et al., 2005). Different types of cells are distributed within tunic (De Leo et al., 1981; Hirose et al., 1994a; Hirose et al., 1994b; Hirose, 2009), playing variable functions, such as accumulation of metabolites and inorganics (Hirose et al., 2006), phagocytosis, pigmentation (Hirose et al., 1999), and defense responses (López-Legentil et al., 2005) in different ascidian species. Cellulose in tunicates is synthesized by a horizontally transferred cellulose synthase gene (*CesA*) (Nakashima et al., 2004; Matthysse et al., 2005; Sagane et al., 2010). A mutant of *CesA* in *Ciona* leads to a variety of phenotypes, including embryonic hatchling defects, notochord alignment, and tail elongation (Sasakura et al., 2005).

MicroRNAs (miRNAs), a class of evolutionally conserved small noncoding RNA molecules with 18–22 nucleotides (Bartel, 2004; Bushati and Cohen, 2007; Leonardo et al., 2012; Bartel, 2018), are commonly identified in eukaryotes and involved in the multiple physiological processes, such as cell differentiation (Foshay and Gallicano, 2009; Sanchez-Vasquez et al., 2019; Yeh et al., 2019), apoptosis (Fujita et al., 2009; Cheng et al., 2018; Sur et al., 2019), and growth and development (Hayashi et al., 2011; Rebustini et al., 2012). In previous studies, we identified 106 known miRNAs and 59 novel miRNAs in *Ciona savignyi*. Among them, *miR4108a*, *miR4000f*, and *miR4018b* were significantly downregulated during metamorphosis (Zhang et al., 2018). The results of *in situ* hybridization showed that the three miRNAs formed a gene cluster and were expressed in the larval trunk; however, the roles of these miRNAs in development, their target genes, and the involved molecular signaling pathways remain elusive.

Based on previous results, this study mainly focused on, and studied, the functions of *miR4018a/4000f/4018b* gene cluster in *C. savignyi*. We found that this gene cluster was required for cellulose synthesis and tunic formation in larvae and metamorphic juveniles. We further identified its target gene *Left–right determination factor* (*Lefty*) and revealed that upregulation of *Lefty* by the decreased expression *miR4018a/4000f/4018b* cluster was essential for this process. Thus, we elucidate a novel microRNA-Lefty molecular pathway that regulates mesenchymal cell differentiation required for the synthesis of cellulose in tunicate species.

## Results

### *miR4018a/4000f/4018b* Cluster was Expressed in the Mesenchymal Cells

**FIGURE 1:**
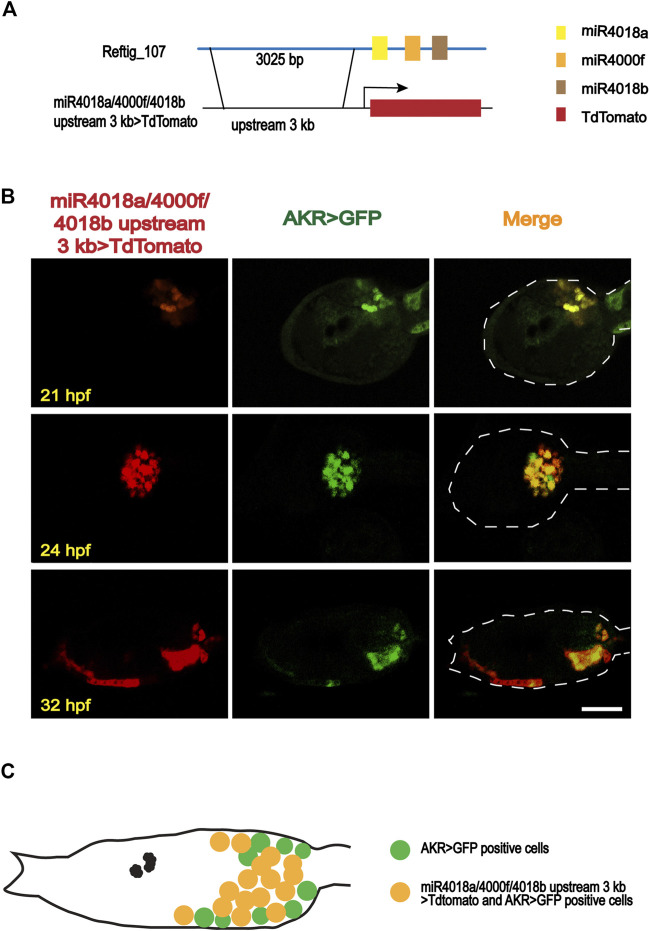
Expression patterns of *miR4018a/4000f/4018b* cluster in *Ciona savignyi*. **(A)** Schematic representation of the regulatory fragment of *miR4018a*/*4000f*/*4018b* cluster. A 3,025-bp upstream fragment of the pre-*miR4018a*/*4000f*/*4018b* cluster was fused with fluorescent protein TdTomato to make a construct. **(B)** *MiR4018a*/*4000f*/*4018b* upstream 3 kb > TdTomato co-expressed with the mesenchymal marker gene *AKR* promoter fusion construct in the larval trunk at 21 h postfertilization (hpf), 24 hpf, and 32 hpf. **(C)** The model of the expression pattern of *miR4018a*/*4000f*/*4018b* cluster in the mesenchymal cells. Statistical data showed that *miR4018a/4000f/4018b upstream 3 kb > TdTomato*-positive cells (*n* = 18 embryos) were all expressed in the *AKR > GFP*-positive cells, suggesting that *miR4018a/4000f/4018b* cluster was expressed in the mesenchymal cell in *C. savignyi*. Scale bar in **(B)**: 50 μm.

Our previous investigation revealed that *miR4018a*, *miR4000f*, and *miR4018b* were consecutively located in scaffold reftig_107 in the genome of the ascidian *C. savignyi*, forming an miRNA cluster (Zhang et al., 2018). Its expression level was significantly downregulated during the larval stage by RNA-seq data (Zhang et al., 2018), indicating its potential roles in larval metamorphosis. We, thus, further validated the expression levels of *miR4018a*, *miR4000f*, and *miR4018b* by qPCR. The results showed that the expression levels of *miR4018a*, *miR4000f*, and *miR4018b* at 42 h postfertilization (hpf), 2 days postattachment (dpa), and 4 dpa were significantly lower than that at 18 and 21 hpf (Supplementary Figure S1). In addition, our previous data showed that all the three members of the microRNAs cluster expressed in a scattered cell population, which was likely mesenchymal cells at the head and trunk part in tailbud embryos (Zhang et al., 2018). To validate the expression tissues, we made a construct containing 3,025 bp upstream of miR4018a/*4000f*/*4018b* fused with TdTomato, *miR4018a/4000f/4018b* upstream 3k *> TdTomato* (Figure 1A), and co-electroporated into *Ciona* eggs with *aldoketoreductase* (*AKR*) promoter-GFP construct, *AKR-*promoter *> GFP* (Tokuoka et al., 2005; Idris et al., 2013; Satoh, 2014; Cao et al., 2019). *AKR* is a mesenchymal marker gene, which is expressed in the mesenchymal cells and becomes a mesenchymal marker at the late larval stage and juvenile stages (Idris et al., 2013). Our results showed that TdTomato signals were scattered in the larval trunk colocalized with AKR signaling at different developmental stages (Figure 1B), validating that the *miR4018a*/*4000f*/*4018b* cluster was indeed expressed in the mesenchymal cell population (Figure 1C).

### Down-Regulation of *miR4018a/4000f/4018b* Cluster was Required for Cellulose Synthesis During *Ciona* Larval Metamorphosis

**FIGURE 2:**
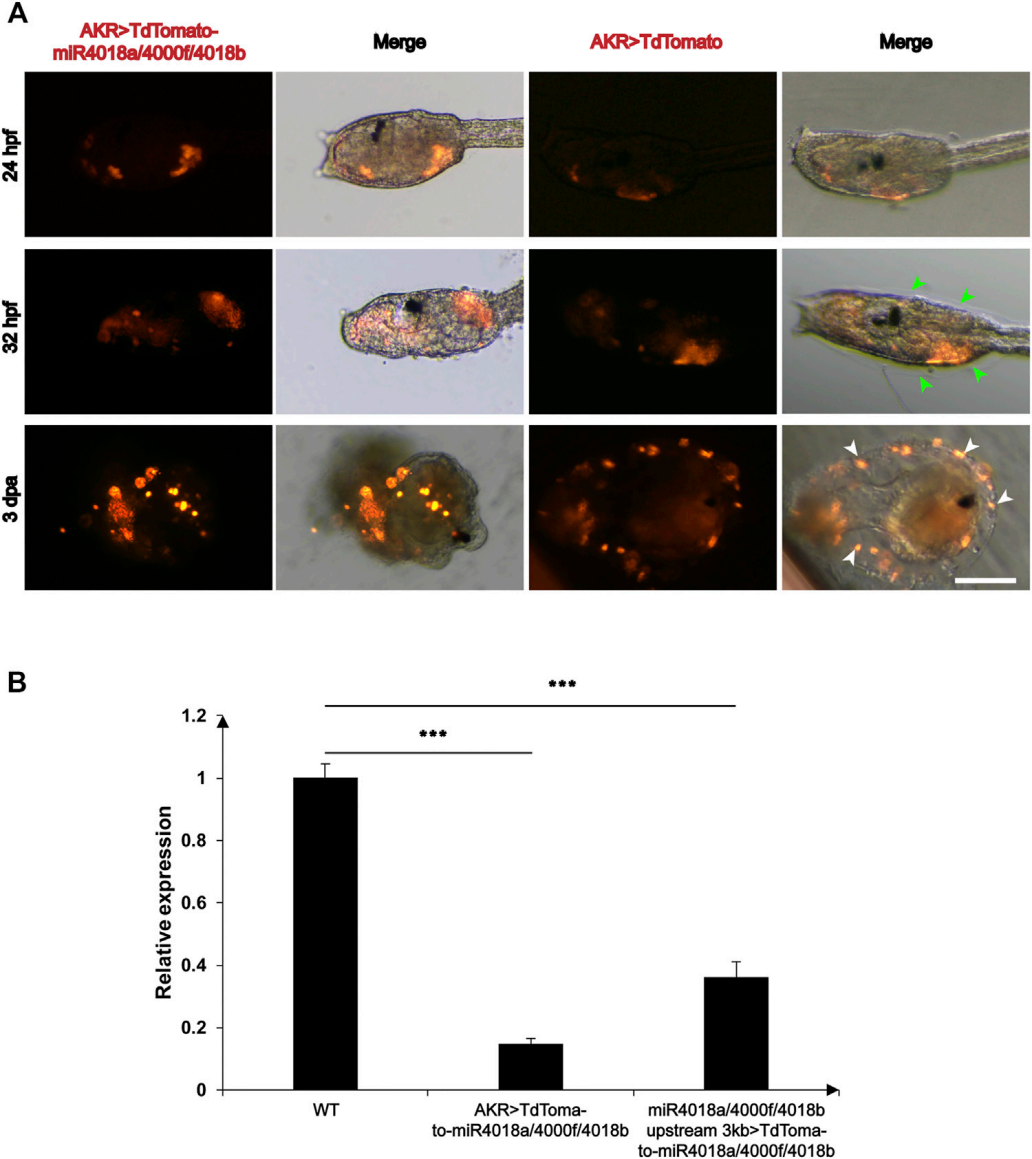
Overexpression of *miR4018a*/*4000f*/*4018b* cluster caused failure of cellulose formation in larvae and loss of tunic cells in the metamorphic larvae. **(A)** *AKR > miR4018a/4000f/4018b-TdTomaoto* and *AKR > TdTomato* (control) were electroporated in the fertilized eggs, respectively. At 32 hpf, the cellulose could be observed surrounding the trunk in *AKR > TdTomato*-expressed larvae (green arrowheads), while in *AKR > miR4018a/4000f/4018b-TdTomaoto-*expressed larvae, cellulose could not be observed. In 3 dpa control larvae, the cells with TdTomato-positive signal could be observed within the tunic (white arrowheads), while in *miR4018a/4000f/4018b* cluster-overexpressed larvae, isolated fluorescent-positive cells could be found surrounding the larvae. Scale bar: 50 μm. **(B)** The expression level of cellulose synthase gene (*CesA*) in control and miRNA-overexpression groups. The results showed that the expression level of *CesA* was decreased significantly at the groups of *AKR* promoter driven and *miR4018a/4000f/4018b* promoter-driven miRNA cluster overexpression compared with the control group. Asterisks (***) represent statistical significance (*p* < 0.001).

We wondered whether *miR4018a/4000f/4018b* cluster is involved in metamorphosis, due to its significant downregulated expression. To validate its roles in mesenchymal cells during larval development, we overexpressed the *AKR* > *TdTomato*-*miR4018a*/*4000f*/*4018b* in *Ciona* larvae using *AKR* driver, and the TdTomato positive signals were detected in mesenchymal cells in both *miR4018a*/*4000f*/*4018b* cluster-overexpressed and control animals at different stages (Figure 2A). We found that the cellulose was significantly decreased and hardly observed in *miR4018a*/*4000f*/*4018b* cluster-overexpressed individuals in swimming and metamorphic larvae (green arrowheads in Figure 2A). The qPCR results showed that the expression levels of three miRNAs in the *miR4018a*/*4000f*/*4018b*-overexpressed group increased significantly compared with the control animals, indicating that the three miRNAs were all overexpressed in *Ciona* larvae successfully (Supplementary Figure S2). In addition, we tracked an individual *Ciona* embryo from the larval stage to juvenile (after metamorphosis) and observed the similar defects of the cellulose synthesis (*n* = 11/17, Supplementary Figure S3). Cellulose synthesis defects were also observed in the larvae overexpressing *miR4018a*/*4000f*/*4018b* cluster by its own driver (*miR4018a/4000f/4018b upstream 3 kb > TdTomato-miR4018a/4000f/4018b*) (*n* = 18/26, Supplementary Figure S4).

Our data also showed the defects of tail absorption in miRNAs overexpression groups. There were 25/31 larvae without the whole tail resorption in miRNA overexpression group, while in the control, 13/29 larvae without the whole tail resorption (white-dashed squares in Supplementary Figures S3 and S4). In addition, we found that miRNA-overexpressed larvae had similar phenotypes in tunic construction with “swimming juvenile” mutant, the tail tunic was narrower than that of wild-type larvae, and the normal fin did not develop (Sasakura et al., 2005).

Since cellulose synthesis in tunicates is mediated by *CesA* (Nakashima et al., 2004; Matthysse et al., 2005; Sagane et al., 2010), and mutant of *CesA* affects the structure of tunic in ascidian (Sasakura et al., 2005), we then investigated the expression levels of *CesA* in *miR4018a*/*4000f*/*4018b* cluster-overexpressed larvae. The results showed that the expression level of *CesA* decreased significantly in miRNA-overexpressed groups compared with that in the control animals (Figure 2B), indicating that *miR4018a*/*4000f*/*4018b* cluster suppresses the expression of *CesA*.

### Overexpression of *miR4018a/4000f/4018b* Cluster Led to the Loss of Tunic Cells

The ascidian tunic is a complex and active tissue that presents mesenchymal characteristics (Hirose et al., 1994b). With the confocal time-lapse imaging, we observed that the mesenchymal cells that migrated and finally presented within the tunic (Figure 2) likely differentiated into the tunic cells. Surprisingly, we found that the floating mesenchymal marker-positive tunic cells were lost in *miR4018a/4000f/4018b* cluster-overexpressed metamorphic larvae, but presented normally within the tunic in control animals (Figure 2A white arrowheads). This suggests that *miR4018a*/*4000f*/*4018b* is involved in the differentiation of mesenchymal cells into tunic cells.

### Identification of Target Genes of *miR4018a/4000f/4018b* Cluster

To reveal the underlying molecular mechanisms, we conducted RNA-Seq using *miR4018a/4000f/4018b*-overexpressed larvae and then sorted 6,902 downregulation expressed genes compared with that in wild-type larvae (Supplementary Figure S5). In addition, we computably predicted 929 target genes based on the seed sequence of *miR4018a/4000f/4018b* cluster by TargetScan (Supplementary Figure S5). Overlapped genes (563) were selected based on RNA-Seq data and TargetScan prediction. Along with the fact that the expression level of *miR4018a/4000f/4018b* cluster was lower at 42 hpf, we chose 47 significantly upregulated genes (log_2_fold <−1) out of 563 candidates. Then we focused on 12 genes based on gene function annotation (Supplementary Table S1).

### *Lefty* was the Target Gene of *miR4018a/4000f/4018b* Cluster

**FIGURE 3:**
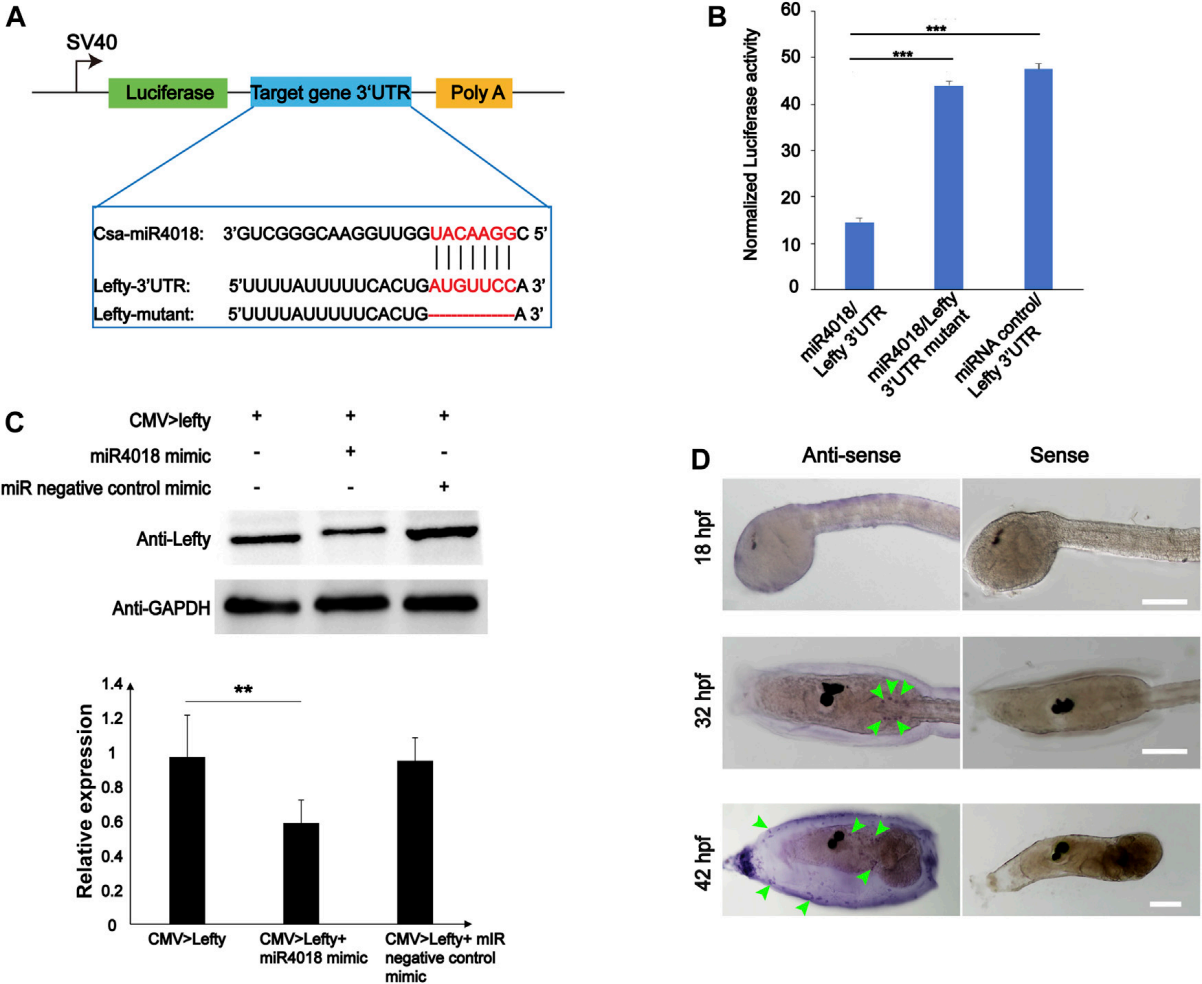
*Lefty* is the target gene of *miR4018*. **(A)** Schematic representation of luciferase reporter constructs carrying the 3′-UTR regions of target genes. *Lefty*-3′ UTR, *Lefty*-3′ UTR mutants were cloned into the downstream luciferase open reading frame of the pmirGLO dual-luciferase vector. Sequence alignments of *csa*-*miR*-*4018* and its predicted target gene and *Lefty*-3′UTR and mutants are displayed in the blue line box. **(B)** Relative luciferase activity in HEK-293T cells cotransfected with pmirGLO-*Lefty* 3′ UTR and miR4018 mimics, pmirGLO-*Lefty* 3′ UTR mutants and *miR4018 mimics*, *Lefty*-3′ UTR mutants and miRNA negative control mimics, respectively. Firefly luciferase values were normalized Renilla luciferase activity. Student’s t-test was used to evaluate the significance of luciferase data. Asterisks (***) represent statistical significance (*p* < 0.001). **(C)** Lefty expression at the protein level in human embryonic kidney 293T cells (HEK293T). GAPDH was used as an internal control in the Western blotting experiments. *CMV > Lefty*, *CMV > Lefty* and *miR4018 mimics*, *CMV > Lefty* and *miRNA negative control mimics* were cotransfected into HEK-293T cells, respectively. Expression level of Lefty in *CMV > Lefty* and m*iR4018 mimi*c group was significantly decreased compared with that in the additional two groups. Asterisks (**) represent statistical significance (*p* < 0.01). **(D)** Expression patterns of *Lefty* detected by w*hole-mount in situ* hybridization. Hybridized signals were specifically detected in a scattered cell population in the trunk in the embryos and larvae at 31 and 42 hpf (green arrowheads). No visible signal was examined in the embryos at 18 hpf. Scale bar: 100 μm.

To validate the target gene of *miR4018a/4000f/4018b* cluster, the 12 genes that were screened out were further experimentally examined through the luciferase report gene assay. We overexpressed miRNAs in cells transfected with luciferase reporter gene constructs containing different target gene 3′UTRs (*Twist* is excluded because it does not have a 3′UTR region binding to miRNA). No significant alteration in luciferase activity has been observed in the groups of *Pmsa3*, *Tll1*, *EeF2k*, *Pdcd4*, *Sele*, *FBXW2*, *Tab1*, *MYOT*, and *TGFR-2* (Supplementary Figure S6). The luciferase activity of miRNA/*F11*-3′UTR was slightly increased compared with the *F11*-3′UTR group; however, it was not very significant (*p* = 0.03, Supplementary Figure S6). Only the *Lefty* gene (Gene ID: ENSCSAVG00000003020) was finally identified as the target of *miR4018a/4000f/4018b* cluster with the fact that the luciferase activity of miR4018/*Lefty* 3′UTR-transfected cells decreased remarkably compared with the miRNA negative control mimic and *Lefty* mutant [removal of *miR4018* binding sites (AUGUUCC)] (Figures 3A, B). Furthermore, we examined the relationship of *miR4018a/4000f/4018b* cluster and *Lefty* in HEK-293T cells by Western blotting. The result showed that the level of Lefty protein decreased remarkably in cells cotransfected with *CMV > Lefty* and *miR4018* mimic compared with that in the group of *CMV > Lefty* only (Figure 3C). These results indicate that *Lefty* is the target gene of the *miR4018a/4000f/4018b* cluster.

### *miR4018a/4000f/4018b* Cluster Regulated Cellulose Synthesis *via* Lefty Signaling

**FIGURE 4:**
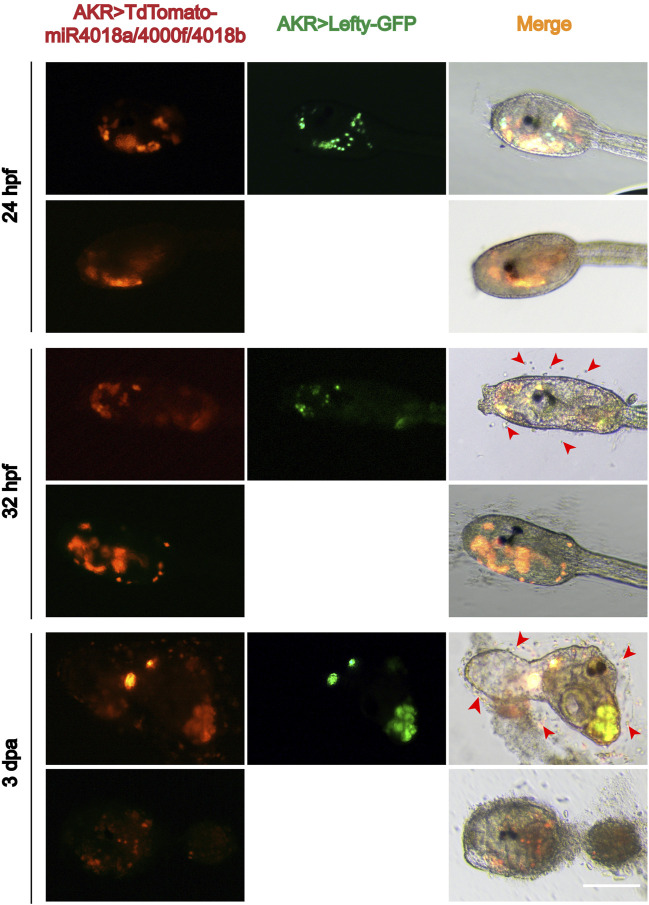
Overexpression of *Lefty* rescue the phenotype in the *miR4018a*/*4000f*/*4018b*-overexpressed larvae. Cellulose and the sporadic tunic cells could be observed in *AKR* > *TdTomato*-*miR4018a*/*4000f*/*4018b* and *AKR* > *Lefty-GFP* coexpressed swimming and metamorphotic larvae at 32 hpf and 3 dpa (red arrowhead) (*n* = 22/35). In *miR4018a/4000f/4018b*-only overexpression group, 25/35 larvae without the tunic. Scale: bar 50 μm.

Single-cell RNA sequencing data showed that *Lefty* is normally expressed in the notochord, B-line mesenchyme, and TVC in *C. savignyi* embryos (Zhang et al., 2020). We detected the expression pattern of *Lefty* in *C. savignyi* larvae at different stages by whole-mount *in situ* hybridization. The results showed that it was specifically detected in a scattered cell population at 31 and 42 hpf, similar to the expression pattern of *miR4018a*/*4000f*/*4018b* cluster (Figure 3D), indicating that *Lefty* was likely also expressed in the mesenchymal cells. The similar expression patterns of *Lefty* with *miR4018a/4000f/4018b* cluster implies that *Lefty* may act as a downstream factor of *miR4018a/4000f/4018b* cluster to regulate cellulose synthesis. To test if Lefty is involved in tunic formation as the target gene of the *miR4018a/4000f/4018b* cluster, we co-overexpressed *miR4018a*/*4000f*/*4018b* and *Lefty* in *Ciona* larvae, in which we found that both tunic and tunic cells were presented (*n* = 22/35, Figure 4). In *miR4018a/4000f/4018b*-only overexpression group, 25/35 larvae were without the tunic (Figure 4). This result implies that *Lefty* could largely rescue the phenotypes in *miR4018a/4000f/4018b*-overexpressed larvae. This experiment demonstrates that *miR4018a/4000f/4018b* regulated cellulose synthesis and tunic formation via Lefty signaling.

## Discussion

The tunic is a multifunctional tissue that provides support and mechanical rigidity. It is mainly composed of cellulose. Tunicates is the only animal group that can synthesize cellulose in the epidermis by a horizontally transferred cellulose synthase gene (*CesA*) (Nakashima et al., 2004; Matthysse et al., 2005; Sagane et al., 2010). In ascidians, the tunic was first observed in the late tailbud stage, and gradually formed a tunic covering the trunk and tail, respectively, during swimming stage. With the development of the metamorphosis of larvae, the tunic of the tail was lost, and a complete tunic was formed in juveniles (Cloney and Cavey, 1982; Stato et al., 1997). During this process, there are free tunic cells between the tunic and epidermis. Studies have shown that tunic cells are derived from mesenchymal cells of the B8.5 and B7.7 cell lineages (Satoh, 2014), and can be divided into different cell types that perform different biological functions, such as accumulation of metabolites and inorganic products (Hirose et al., 2006), pigmentation (Hirose et al., 1999), defense responses (López-Legentil et al., 2005), and phagocytosis (Li et al., 2020). In addition, tunic formation requires the presence of tunic cells. Some free tunic cells contain and deposit molecules, which modify the larval tunic, so they may be involved in the formation of tunic in the ascidian embryos (Bishop et al., 2010). Tunic cells can migrate during the whole embryonic development process, form a large number of aggregates, and migrate to the outer surface of the embryo to perform their functions (Bishop et al., 2010).

In vertebrates, bone morphogenetic protein (Chen et al., 2019), fibroblast-binding factors (FGFs) (Sun and Stathopoulos, 2018), transforming growth factor beta (Hao et al., 2019), Wingless-type/β-catenin (Jarvinen et al., 2018; Chen et al., 2019), and other signaling pathways are involved in the differentiation of mesenchymal stem cells into diverse cell types, such as chondrocytes (Yang et al., 2018), odontogenic cells (Cui et al., 2018), and muscle cells (De Bari et al., 2003; Kumar et al., 2017). Also, *miR-145* mediates the differentiation of mesenchymal cells into smooth muscle cells (Yeh et al., 2019), and mesenchymal stem cell-derived exosome *miR-223* regulates neuronal cell apoptosis (Wei et al., 2020). In ascidian, it has been reported that FGF, pmRACK1, Tbx6, and twist-like signaling play a center role in the fate determination from mesenchymal cell to other organs, such as the muscle, somatic, gonad, nerve cord, tunic, and heat (Tokuoka et al., 2005; Kim et al., 2007; Tatzuke et al., 2012; Kumano et al., 2014). In this study, we showed that a novel ascidian miRNA cluster (*miR4018a/4000f/4018b*) was expressed in the mesenchymal cells of larvae and is downregulated during larval metamorphosis. Overexpression of *miR4018a/4000f/4018b* cluster caused the failure of cellulose synthesis in swimming larvae and loss of tunic in metamorphic juveniles.

We speculated that *miR4018a/4000f/4018b* cluster might be responsible for tunic cell development. Overexpression of this miRNA cluster affected its downstream gene expression and inhibited the differentiation of mesenchymal cells into tunic cells. Defective tunic cell specification may have an impact on epidermal cells and eventually disrupted the expression of the essential genes (e.g., *CesA*) related to cellulose synthesis. In order to explore the molecular mechanism, we conducted a screening based on RNA-Seq and TargetScan prediction to find the target genes of miR4018a/4000f/4018b. *Lefty* was finally identified as the target after screening and luciferase assay. *Lefty* is a member of the TGF-β subfamily, and its encoded protein can regulate stem cell differentiation by competitively binding TGF-β receptors (Tabibzadeh and Hemmati-Brivanlou, 2006). *Lefty* is known to regulate the balance of cell pluripotency and cell differentiation through the *Lefty*/*Nodal* signaling pathway (Dai et al., 2016; Li et al., 2018; Zhang et al., 2019). In addition, Lefty can also cooperate with Smad (mothers against decapentaplegic homolog), Wnt, and the transcriptional factor octamer-binding factor 3/4 to support cell stemness (Tabibzadeh and Hemmati-Brivanlou, 2006). We further showed that *Lefty* overexpression could rescue the defects of tunic formation and loss of tunic cells caused by *miR4018a/400f/4018b* overexpression, confirming that miRNA cluster-Lefty pathway is required for the differentiation of *Ciona* mesenchymal cells into tunic cells and cellulose synthesis.

Our study also found that miRNA overexpression caused a more severe tunic-deficiency phenotype than that in *CesA* mutant (Sasakura et al., 2005). It is possible that overexpression of miRNA cluster regulates additional targets that affect tunic synthesis process, such as extracellular matrix components.

In conclusion, we report a novel *miR4018a/4000f/4018b* cluster-Lefty pathway that plays an important role in the differentiation of mesenchymal cells into tunic cells and synthesis of cellulose, providing insights into the understanding of the mesenchymal cell development and differentiation, and formation of tunic structure in tunicates.

## Methods

### Adult Animals, Fertilization, and Electroporation

The ascidian *C. savignyi* adults were collected from the eastern coast of Qingdao (Shandong, China) and maintained in a seawater tank for 3 days under constant light to accumulate gametes in the laboratory. The eggs and sperm were obtained from different individuals and then fertilized in the seawater for 5 min. After fertilization, eggs were dechorionated, electroporated, and then cultured at 16°C for development. The electroporation was performed as the previously described protocol (Lu et al., 2020).

### Promoter Cloning and Plasmid Construction

The PCR of a promoter was performed with the Phanta Max Super-Fidelity DNA Polymerase (Vazyme, Nanjing, China). The PCR product was purified by GeneJET Gel Extraction Kit (Thermo Scientific, Waltham, MA, USA) and then ligated into the vector containing the reporter gene GFP or TdTomato. All constructs were generated using the one ClonExpress MultiS One Step Cloning Kit (Vazyme, Nanjing, China) and confirmed by sequencing. The sequences of primers used for plasmid construction are shown in Supplementary Table S2.

### Quantitative PCR

Two types of samples were used for quantitative PCR (qPCR). One was the embryos/larvae at the different stages (18, 21, 25, 42 hpf and 2, 4 dpa), another was the wild-type and *miR4018a*/*4000f*/*4018b*-overexpressed larvae collected at 31 hpf. The total RNA was extracted with RNAiso Reagent (Takara). All cDNAs were synthesized using the miRNA First Strand cDNA Synthesis Kit (by stem-loop) (Vazyme, Nanjing, China). qPCR amplification was performed using the miRNA Universal SYBR qPCR Master Mix Kit (Vazyme, Nanjing, China) on the Roche Applied Science LightCycler^TM^ 480 Real-Time PCR System. According to the supplier’s protocol, the reaction was carried out with the following conditions: 95°C for 5 min, 40 cycles of 95°C for 10 s and 60°C for 30 s, 95°C for 15 s, 60°C for 60 s, and 95°C for 15 s. Data were calculated using the 2^−ΔΔCt^ method, and statistical analyses were performed using paired Student’s t-tests. A value of *p* < 0.05 was considered statistically significant.

### Whole-Mount *in situ* Hybridization

The ascidian larvae were fixed in 4% paraformaldehyde for 2 h at RT. *In situ* hybridization was performed as previously described (Zhang et al., 2018). Labeled antisense RNA probes were transcribed from linearized DIG RNA Labeling Mixture (Roche America) according to the manufacturer’s instructions. The *Lefty* probe size is 780 bp, and the sequences of primers are as follows: forward primer, 5′ TTA​TCG​TCC​TGT​TCC​TCG​CA 3′ and reverse primer, 5′ GCT​GGT​TCC​TTC​ACG​TTT​GT 3′.

### RNA-Seq and the Target Gene Prediction

The wild-type and *miR4018a*/*4000f*/*4018b* cluster-overexpressed larvae were collected at 32 hpf, and the total RNA was extracted and sent for RNA-seq. The gene libraries were constructed according to the NEB common library construction method, and the original data was formed by Illumina sequencing. Clean reads for subsequent analysis were obtained after filtration of original data, sequencing error rate check, and GC content distribution check. Clean reads after quality control were compared with the genome for gene annotation, and the sequencing of the reference transcription construction library was completed. Differential gene enrichment analysis was performed using NovoMagic (https://magic.novogene.com/).

The target genes of miR4018a, 4000f, and 4018b were predicted using TargetScan. 3′UTRs of *C. savignyi* mRNAs obtained from the ENSEMBL database were used as potential target sequences. The predicted targets with downregulated expression after miR4018a/4000f/4018b overexpression were collected for further analysis (Zhang et al., 2018).

### Cell Culture and Luciferase Assay

Human embryonic kidney 293T cells (HEK293T) were cultured in the following conditions: Dulbecco’s modified Eagle’s medium (DMEM, Invitrogen, USA) supplemented with 10% fetal bovine serum (Gibco, USA), penicillin (100 U/ml), and streptomycin (100 μg/ml) grown in a humidified atmosphere of 5% CO_2_ at 37°C.

The wild-type 3′UTR fragments of 12 candidate target genes containing predicted cas-*miR4018* or cas-*miR4000f* seed sites were amplified by PCR using cDNA as templates. The PCR products were cloned into the firefly luciferase coding region of the pmirGLO plasmid to construct reporters for luciferase assays. For the luciferase assay, 6 h before cell transfections, HEK293T cells were passaged in 96-well plates with 3 × 10^4^ cells per well. Then the cells were cotransfected with 0.1 μg of luciferase reporter plasmid and 100 nM miRNA mimics. Twelve hours after transfection, a fresh medium was placed into each well. Forty-eight hours after transfection, firefly and Renilla luciferase activities were measured using the dual-luciferase reporter assay system (Beyotime, China), and relative reporter activity was normalized to Renilla luciferase activity. The data statistical analyses were performed using paired Student’s t-tests. Experiments were performed in triplicate.

### Western Blot

The total protein (20 μg) was used for 10% SDS-polyacrylamide gel electrophoresis at 150 V for 1 h and then transferred onto polyvinylidene fluoride (PVDF) membranes in Western transfer buffer at 60 mA for 3 h in an ice bath. Nonspecific binding sites were blocked with 5% fat-free powdered milk in Tris-buffered saline plus Tween 20 [0.2% (vol/vol)] (TBST) (blocking solution) at room temperature (RT) for 2 h. After washing three times with TBST (each for 15 min), the membranes were incubated overnight at 4°C with primary Lefty antibody (Lefty antibody: TBST = 1:1,000) (Abcam Chain). After the incubation, the PVDF membranes were washed three times and then incubated at RT for 2 h with the horseradish peroxidases (HRP)-conjugated secondary antibody. After washing three times with TBST (each for 15 min), proteins were detected using enhanced chemiluminescence (Advansta, Menlo Park, CA, USA). Quantification was performed using the Quantity One program (Bio-Rad). The experiments were performed three times in triplicate. Gray scale analysis was performed using ImageJ, and data statistical analyses were performed using paired Student’s t-tests.

## Data Availability

The data presented in the study are deposited in the SRA repository, accession number PRJNA790731.
