# Supplementary material for: A microRNA Cluster-Lefty Pathway is Required for Cellulose Synthesis During Ascidian Larval Metamorphosis
Source: Front Cell Dev Biol. 2022 Mar 15;10:835906. doi: 10.3389/fcell.2022.835906 (PMC8965075; doi:10.3389/fcell.2022.835906)
Supplement: Supplementary file 1 [file DataSheet1.pdf]

Supplementary data

A microRNA cluster-Lefty pathway is required for cellulose synthesis for ascidian larval metamorphosis

Xueping Sun[a], Xiaoming Zhang[a], Likun Yang[a], Bo Dong[a,b,c,*]

a. Sars-Fang Centre, MoE Key Laboratory of Marine Genetics and Breeding, College of Marine Life Sciences, Ocean University of China, Qingdao 266003, China.
b. Laboratory for Marine Biology and Biotechnology, Qingdao National Laboratory for Marine Science and Technology, Qingdao 266237, China
c. Institute of Evolution & Marine Biodiversity, Ocean University of China, Qingdao 266003, China

[*]To whom correspondence should be addressed. Email: bodong@ouc.edu.cn

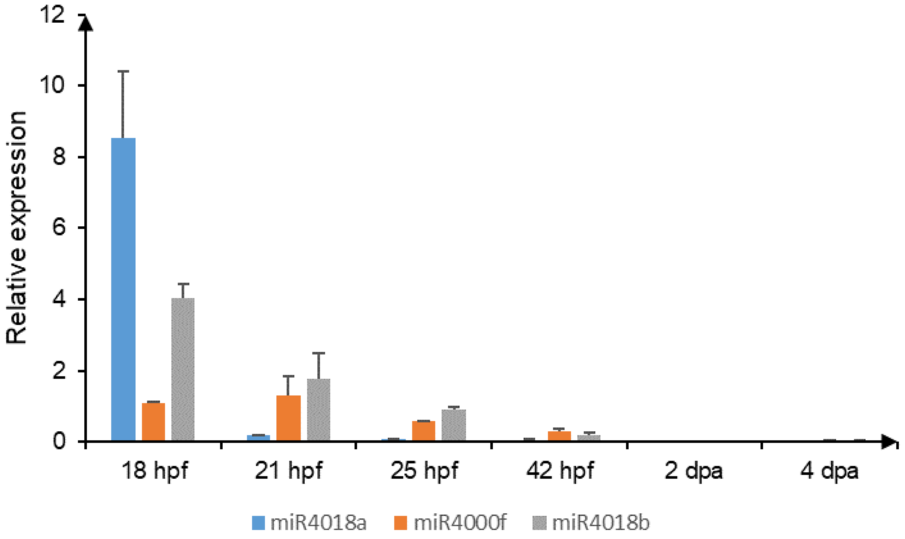

Supplemental figure 1: The expression levels of *miR4018a, miR4000f,* and *miR4018b* were examined at 18 hpf, 21 hpf, 25 hpf, 42 hpf, 2 dpa, and 4 dpa by qPCR. The relative expression levels of these miRNAs at later larvae and juveniles were significantly lower compared with that at 18 hpf and 21 hpf.

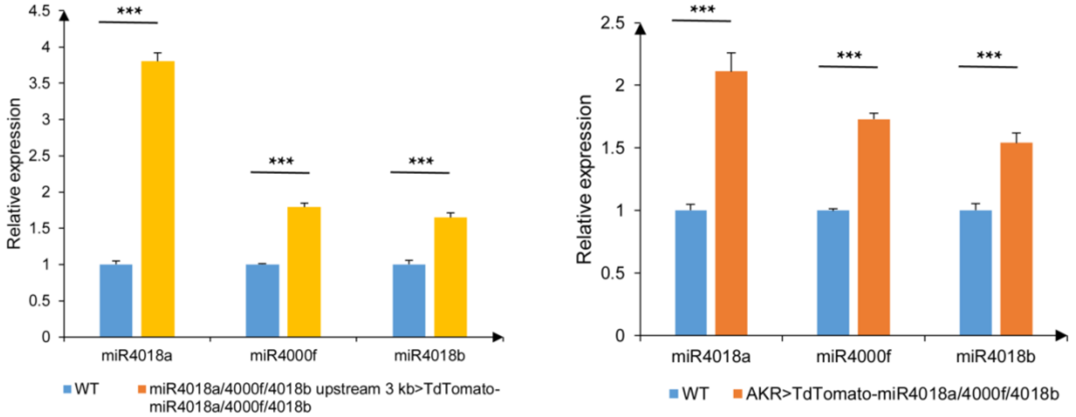

Supplemental figure 2: The expression levels of *miR4018a*, *miR4000f*, and *miR4018b* were measured in control and the overexpressed ascidian larvae by qPCR. **(a)** The expression levels of *miR4018a*, *miR4000f*, and *miR4018b* in *miR4018a/4000f/4018b>TdTomato-miR4018a/4000f/4018b* overexpressed and control larvae. **(b)** The expression levels of *miR4018*, *miR4000f*, and *miR4018b* in AKR>TdTomato-*miR4018a/4000f/*4018b overexpressed and control larvae. Asterisks (***) represent statistical significance ($p < 0.001$).

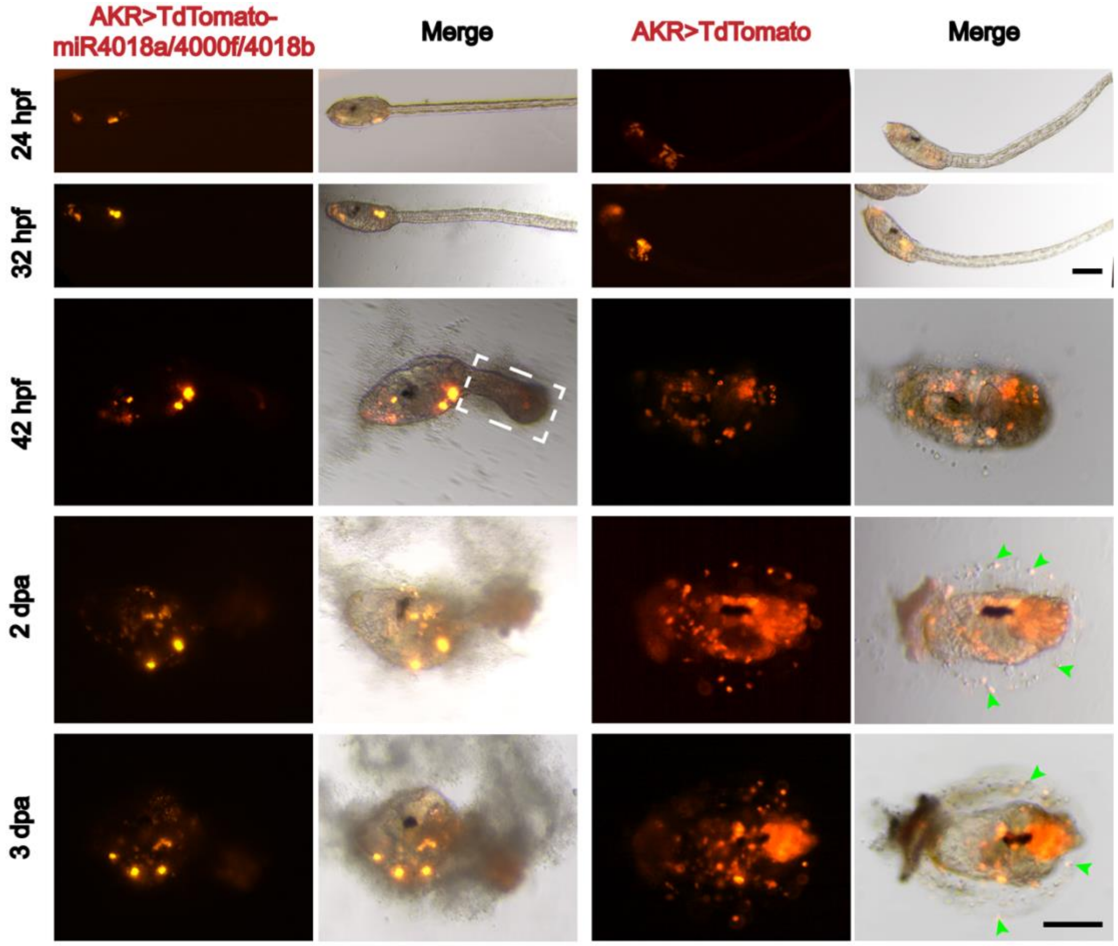

Supplemental figure 3: Development of ascidian larval with overexpression of *miR4018a/4000f/4018b* cluster. Overexpression of *miR4018a/4000f/4018b* was driven by *AKR* promoter. The time lapse images were captured from one individual larval at the different developmental stages. The tunic could be observed in the control (green arrowhead) (n = 9/12) but not in *miR4018a/4000f/4018b* cluster overexpressed larvae (n = 11/17). White-dashed square indicates the failure of tail absorption in miRNAs overexpression larvae. Scale bar 100 μm.

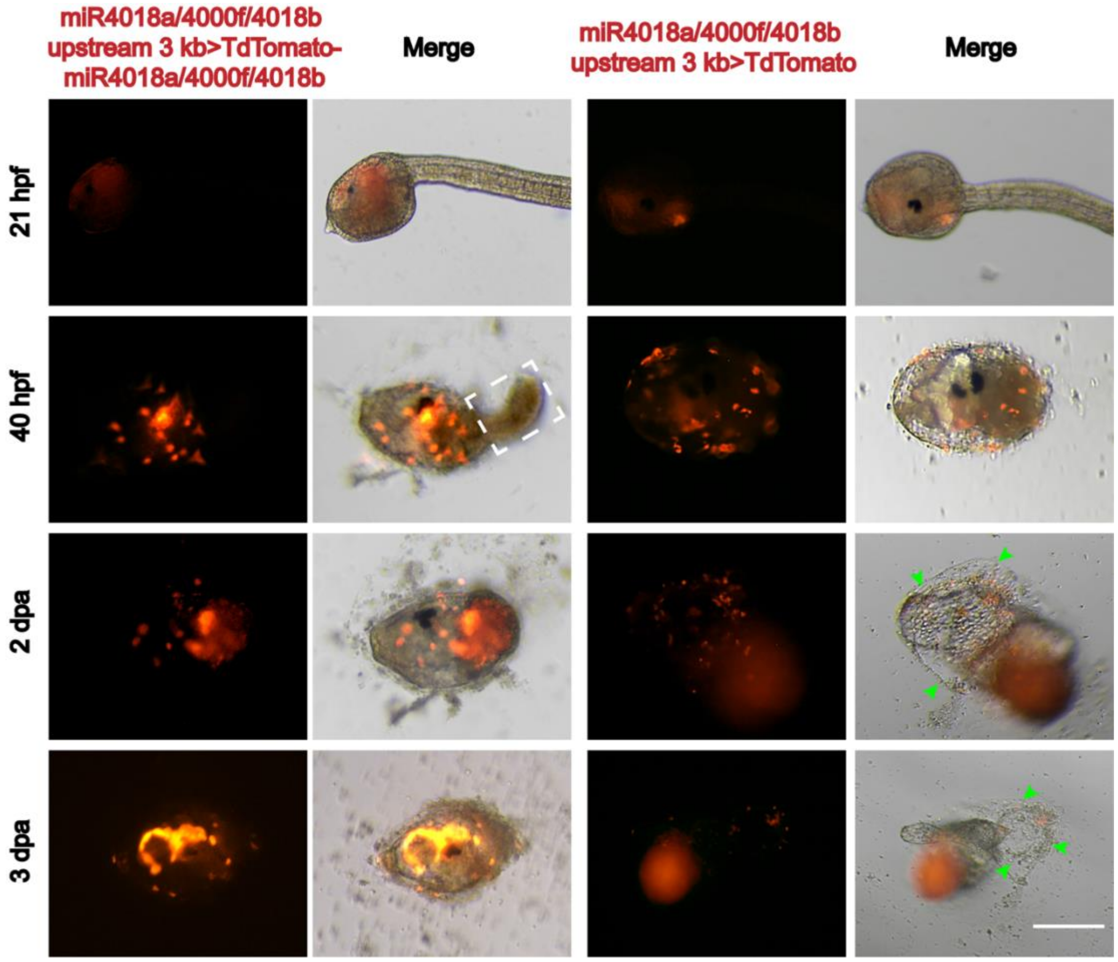

Supplemental figure 4: Development of ascidian larval with overexpression of *miR4018a/4000f/4018b* cluster. Overexpression of *miR4018a/4000f/4018b* was driven by *miR4018a/4000f/4018b* upstream 3 kb fragment. The time lapse images were captured from one individual larval at the different developmental stages. The tunic could be observed in the control (green arrowhead) (n = 21/30) but not in *miR4018a/4000f/4018b*-cluster overexpressed larvae (n = 18/26). White-dashed square indicates the defect of tail absorption in miRNAs overexpression larvae at 40 hpf. Scale bar 100 μm.

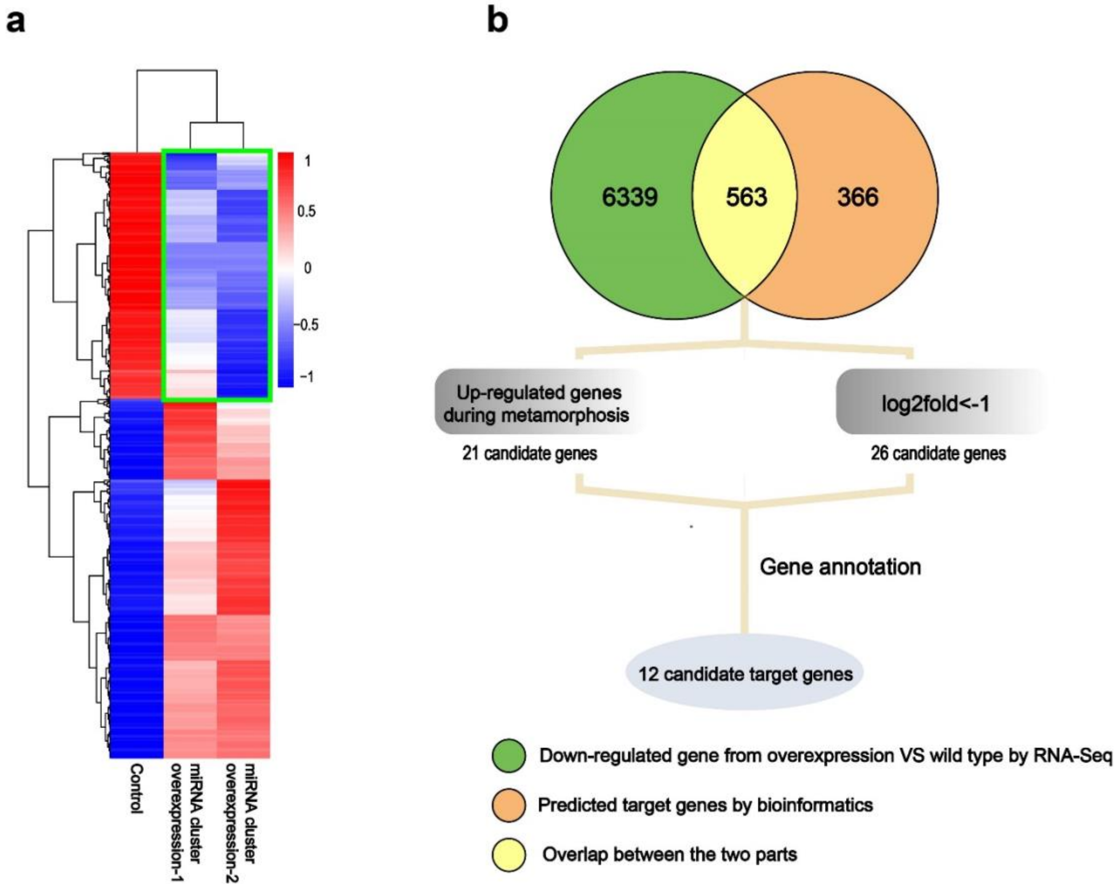

Supplemental figure 5: Target gene screening for *miR4018a/4000f/4018b* cluster. (**a**) the heatmap of genes from RNA-seq. The genes in green square were down-expressed ones in *miR4018a/4000f/4018b* cluster-overexpressed larvae. (**b**) The narrow-down procedures of target gene screening. Venn diagram shows the number of miRNA cluster targets and their overlapping spots predicted by the two strategies (RNA-Seq and TargetScan prediction). Then 563 overlapped genes with up-regulation at 42 hpf, or met the standard (log_2fold<-1) were sorted out. Finally, 12 target genes were picked up through gene annotation.

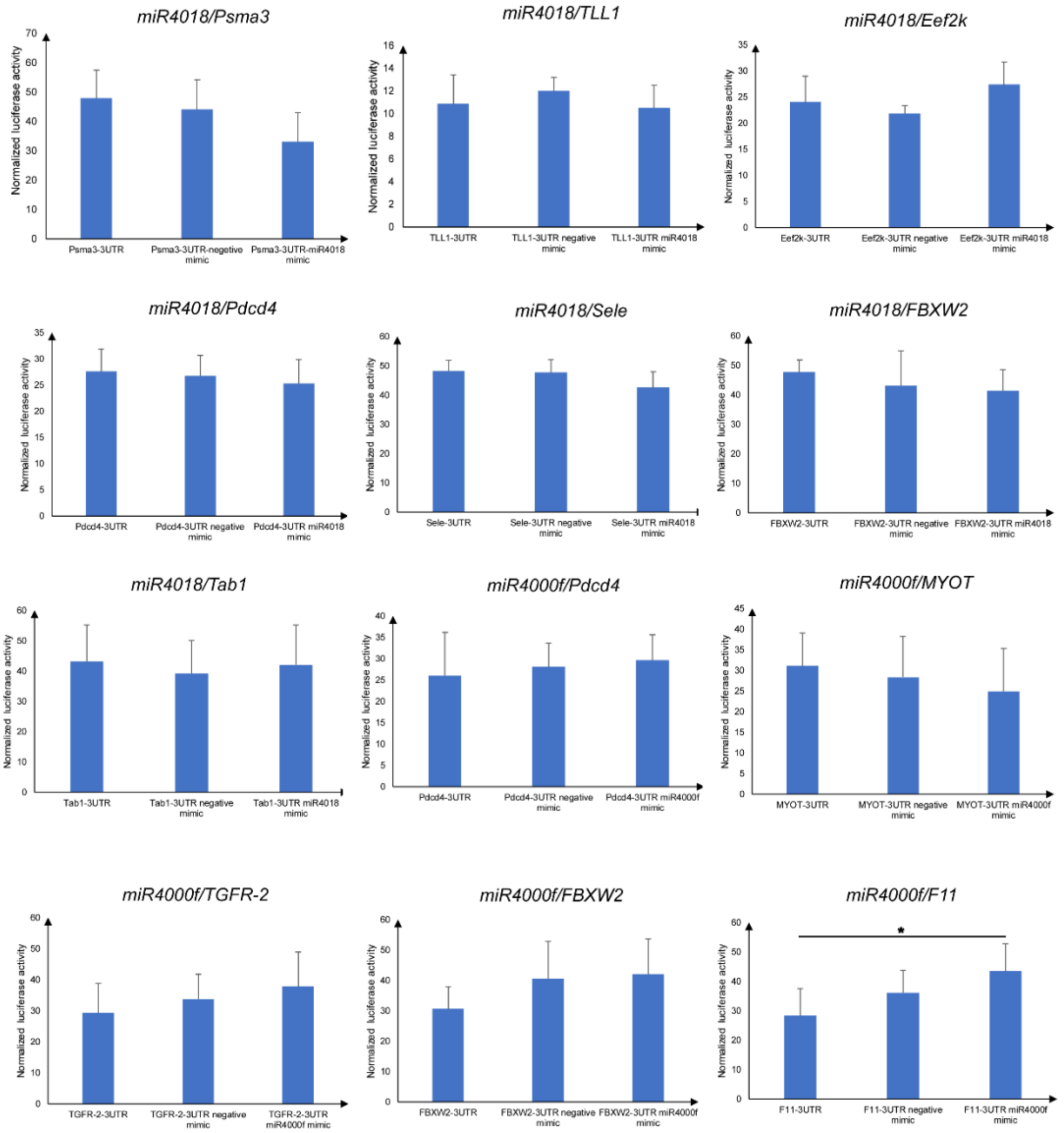

Supplemental figure 6: Luciferase reporter gene detected the target genes of miRNAs. Relative luciferase activity in HEK-293T cells co-transfected with pmirGLO-target-3′UTR and miRNAs mimics, miRNA negative control mimics, respectively. Firefly luciferase values were normalized Renilla luciferase activity. Student's t-test was used to evaluate the significance of luciferase data. Asterisk (*) represents statistical significance ($p = 0.03 < 0.05$).

Supplemental table 1: The predicted target genes of *miR4018* and *miR4000f*.

miRNA family	Gene ID	Description
>>csa-miR-4000f	ENSCSAVG00000000758	Coagulation factor XI OS=Mus musculus GN=F11 PE=2 SV=2
>>csa-miR-4000f	ENSCSAVG00000002854	Myotilin OS=Homo sapiens GN=MYOT PE=1 SV=2
>>csa-miR-4000f	ENSCSAVG00000006972	TGF-beta receptor type-2 GN=TGFR-2
>>csa-miR-4018	ENSCSAVG00000000720	Proteasome subunit alpha type-3 OS=Rattus norvegicus GN=Psma3 PE=1 SV=3
>>csa-miR-4018	ENSCSAVG00000001784	Tolloid-like protein 1 OS=Homo sapiens GN=TLL1 PE=1 SV=1
>>csa-miR-4018	ENSCSAVG00000002050	Eukaryotic elongation factor 2 kinase OS=Rattus norvegicus GN=Eef2k PE=1 SV=1
>>csa-miR-4018	ENSCSAVG00000011382	Protein twist OS=Drosophila erecta GN=twi PE=3 SV=1
>>csa-miR-4018	ENSCSAVG00000006947	E-selectin OS=Mus musculus GN=Sele PE=2 SV=1
>>csa-miR-4018	ENSCSAVG00000001387	TGF-beta-activated kinase 1 and MAP3K7-binding protein 1 OS=Mus musculus GN=Tab1 PE=1 SV=2
>>csa-miR-4018	ENSCSAVG00000003020	Transforming growth factor beta-1 OS=Mustela putorius furo GN=TGFB1 PE=2 SV=1
>>csa-miR-4018、>>csa-miR-4000f	ENSCSAVG00000005003	Programmed cell death protein 4 OS=Rattus norvegicus GN=Pdcd4 PE=1 SV=2
>>csa-miR-4018、>>csa-miR-4000f	ENSCSAVG00000001347	F-box/WD repeat-containing protein 2 OS=Homo sapiens GN=FBXW2 PE=1 SV=2

Supplemental table 2: The primers sequence of plasmids used in this study.

**Primer name**	**Sequence**
miR4018a/4000f/4018b upstream 3 kb-F	GGTACCTTCGTGCGGGTACTCAAATG
miR4018a/4000f/4018b upstream 3 kb-R	GGATCCTGTTAATACCGGCCTGTTCC
TdTomato-F	ATGGTGAGCAAGGGCGAGG
TdTomato-R	TTACTTGTACAGCTCGTCCATGCCG
AKR 2 kb-F	CAAAACTATTGTAGACCTTTTCTCG
AKR 2 kb-R	AAAGTAGAAATGCATCACTAAACTG
precursor-miR4018a/4000f/4018b-F	CGGCTGGCGCTCGGAACT
precursor-miR4018a/4000f/4018b-R	CGACAAACTGGATTCCGTGCTAAAG
miR4018a-rt-F	GTCGTATCCAGTGCAGGGTCCGAGGTATTCGCACTGGATACGACCATCCC
miR4000f-rt-F	GTCGTATCCAGTGCAGGGTCCGAGGTATTCGCACTGGATACGACCCGGCC
miR4018b-rt-F	GTCGTATCCAGTGCAGGGTCCGAGGTATTCGCACTGGATACGACCCAGCC
miR4018a-qpcr-F	CGCGGAACATGGTTGGAAC
miR4000f-qpcr-F	CGTGAAACTTCGCTGGAACA
miR4018b-qpcr-F	CGAGGAACATTCTGTGGAACG
CesA-qpcr-F	CTGCTGGATTGTGTAGAA
CesA-qpcr-R	GATGACGAGGAAGGTAAC
Lefty-F	ATGACGTCACAAGCATCCAACG
Lefty-R	CACCTCATAACCACTATCTAGCGCA
Lefty-insitu-F	TTATCGTCCTGTTCCTCGCA
Lefty-insitu-R	GCTGGTTCCTTCACGTTTGT
Lefty-3UTR-F	CTAGCTAGCATTGGATTCTTGAACCGGCC
Lefty-3UTR-R	CTAGTCTAGACGCGTGGGGTAGTTTATTGG
Leftymu-F1	TTTTTTTGTTTTTATTTTTCACTGAATAAACTACCC
Leftymu-R1	TCACGCGTGGGGTAGTTTATTCAGTGAAAAATAA
Lefty-cds-F	ATGACGTCACAAGCATCCAACG
Lefty-cds-R	AATCACGCGTGGGGTAGTTTATT
